# Assessing the flow to low-income urban areas of conservation and environmental funds approved by California’s Proposition 84

**DOI:** 10.1371/journal.pone.0211925

**Published:** 2019-02-07

**Authors:** Ian P. Davies, Jon Christensen, Peter Kareiva

**Affiliations:** 1 School of Environmental and Forest Sciences, University of Washington, Seattle, Washington, United States of America; 2 Institute of the Environment and Sustainability, University of California Los Angeles, Los Angeles, California, United States of America; Universitat Jaume I, SPAIN

## Abstract

Government funding accounts for a large proportion of conservation and environmental improvements, and is often the result of citizen votes on state ballot measures. A key concern surrounding public investments in the environment is whether that funding serves lower-income communities, which are often the communities of greatest need. We applied three statistical methods to analyze the spatial distribution of conservation funding derived from California’s Proposition 84, which distributed nearly $4 billion across California between 2006 and 2015. First, we used hurdle models to ask if income, population density, urban coverage, or pollution could explain receipt of grants or magnitude of funding. Second, we compared the income levels of funded and unfunded communities for each chapter of the proposition. Finally, we examine two sections of the proposition that were intended to fund parks around the state and compare the attributes of funded and unfunded communities. Proposition 84 offers lessons for environmental legislation and future research. While there were general tendencies for more funding to flow to poor areas and areas with pollution problems, the language in Proposition 84 as a whole was vague with respect to the funding of disadvantaged areas, and as a result the targeting of these areas overall was at best modest. However, when enabling legislation (AB 31) defined specific “metrics of disadvantage” that had to be met by communities to receive funds from some sections of Proposition 84, the funds did flow much more selectively to poorer communities. This suggests that future ballot measures should be very explicit in their language if they want to promote equity in conservation investments, and that future research should investigate the extent to which technical workshops and outreach could further increase the number of funded grant proposals from low-income communities.

## Introduction

In the United States, ballot measures have become one of the largest funding mechanisms for public investment in conservation and environmental improvements. In the last decade, $40 billion of funding has been approved through ballot measures for conservation alone [1]. When a ballot measure or funding program is approved, headlines typically announce a victory for the environment and a victory for conservation [2]. However, critics of these ballot measures have pointed out that the funding often fails to address social inequities in environmental protection and access to parks and natural areas [3,4]. Although minority groups such as Hispanics and African Americans consistently vote in favor of environmental measures, if these measures are seen to reinforce social inequality, this key support for conservation and environmental protection may be lost [5]. Thus it is increasingly important to examine conservation and environmental funding from an environmental justice perspective and ask how well low-income and high-need communities are served [6,7]. In their analysis of the 1996 Proposition K in Los Angeles, Wolch et al. found that park funding from this public ballot measure often compounded existing inequalities in park access by funding park improvements rather than investments in new properties [3]. Other studies suggest that funding often lacks a focus on structural inequity in park access; that is, urban residents tend to use park space more intensively than their rural and suburban counterparts, a fact that is ignored when measuring access by park space per unit area rather than per active user [3,8,9]. Following the money is important because if there are particular social or environmental needs that are not receiving funds, adjustments could be made for future policy. Yet to our knowledge, such a quantitative analysis has never been performed for a state ballot measure.

Throughout California’s history, a number of ballot measures have been passed with the intention to fund environmental projects. These range from local measures like Proposition K in Los Angeles to the statewide Propositions 12 and 13 which all, in varying ways, intended to channel public funds to increasing park space and improving access. Minorities tend to live in cities with less local fiscal capacity to spend on parks, and for cities in California, public funds from state legislation and ballot measures have become a viable model for building infrastructure [10]. Large environmental nonprofits play an important role in this process by helping to craft these measures through political partnerships and then donating to them so that they are passed [11]. While successful in passing park measures, some have expressed concern that nonprofits whose concern is habitat protection will prioritize green spaces on the edges of cities, rather than in the urban core where few have access to open spaces [10]. When these decisions are made in writing measures and awarding grants, they can lead to a distribution of park resources that is not equitable for the communities most in need.

### An Overview of Proposition 84

Proposition 84, a general obligation bond, was passed in 2006 and at the time represented the largest state ballot measure in the United States for environmental protection. Notably, it was carried largely with support from California Latinos, who voted 84% in favor versus just 45% from non-Hispanic white voters [12]. Proposition 84 authorized $5.4 billion in spending on water quality and supply, natural resource protection, and urban greening in high-need areas–a wide breadth, leading some to criticize the measure for lacking clarity and accountability [13,14]. Others criticized the explicit earmarking of funds to specific groups, like the San Joaquin River Conservancy, as evidence of too much sway from environmental donors [15].

Proposition 84 funded projects through a competitive grant process. Under “general provisions” the text of Proposition 84 specified the following social priorities: “assistance to communities with contaminated sources of drinking water” and “revitalizing our communities and making them more sustainable by investing in … local parks and urban greening” [16]. The $5.4 billion was then divided by project type into nine distinct chapters, each with its own grant criteria for funding (Table 1).

**Table 1:** Proposition 84 fund allocation from 2006–2015. Grants with local impacts are those projects we determined to have identifiable “on-the-ground” impacts in local communities, as opposed to planning and technology grants and large regional projects where local community impacts could not be identified. Chapter 1 of the ballot measure details general provisions of the proposition but has no funding tied to it, so it is excluded from this analysis.

Chapters	Funding Authorized (millions)	Funding Awarded (in millions of dollars and number of grants)		
		Grants with local impact	Grants for which impacts are not readily assigned to particular locations	All grants
2: Safe Drinking Water and Water Quality Projects	$1,525	$143 173	$1,003 796	$1146 969
3: Flood Control	$800	$182 36	$411 342	$594 378
4: Statewide Water Planning and Design	$65	$0 0	$64 17	$63 17
5: Protection of Rivers, Lakes, and Streams	$928	$524 594	$132 399	$656 993
6: Forest and Wildlife Conservation	$450	$299 221	$26 30	$325 251
7: Protection of Beaches, Bays, and Coastal Waters	$540	$161 249	$140 266	$301 515
8: Parks and Nature Education Facilities	$500	$236 571	$67 118	$303 689
9: Sustainable Communities and Climate Change Reduction	$580	$419 308	$102 256	$521 564
Total	$5,388	$2,152 2152	$1,946 2224	$3,909 4376

While Proposition 84 was intended to fund many different types of projects around the state, two of the eight chapters in their subchapters contained language that could be interpreted, in part, as serving an environmental justice or urban-focused agenda by either specifically prioritizing “disadvantaged” (low-income) communities, communities with pollution burdens, or those undergoing population growth. The chapters did so, however, with language that differed in its specificity.

In particular, Chapter 2 for “Safe Drinking Water and Water Quality Projects” directed $1.18 billion towards water quality projects with priority given to “projects that address chemical and nitrate contaminants, other health hazards and by whether the community is disadvantaged or severely disadvantaged.” *[“Disadvantaged communities” have median household incomes less than 80% of the statewide average*, *“severely disadvantaged communities” less than 60%*.*]* [16]. Chapter 2 prioritized communities along six criteria, including those stated above, and stated that at least one must be met.

Chapter 8 for “Parks and Nature Education Facilities” stated: “The Department of Parks and Recreation shall include the following goals in setting spending priorities … The expansion of the state park system to reflect the growing population and shifting population centers and needs of the state” [16].

Chapter 9 for “Sustainable Communities and Climate Change Reduction” projects stated: “Acquisition and development of new parks and expansion of overused parks and recreation areas that provide park and recreational access to underserved communities shall be given preference.” And “creation of parks in neighborhoods where none currently exist shall be given preference” [16]. This section was enabled with more specific criteria through AB 31, the “Statewide Park Development and Community Revitalization Act of 2008”, which further directed funding for “the acquisition and development of parks and recreation areas and facilities in the communities that are currently least served by park and recreation facilities by emphasizing the creation of park space and recreational opportunities and the expansion of park accessibility to underserved communities.” *[A “critically underserved community” has <3 acres of usable parkland per 1*,*000 residents or is “disadvantaged” (see above)]* [17].

For AB 31, the process of awarding these competitive grants was overseen by the Department of Parks and Recreation with quantitative criteria written by politicians and “diverse allies,” including Los Angeles social justice organization The City Project [18]. In their application guide, the department included a scoring rubric that awarded more points for new parks in areas where there are no existing parks, for applicants holding meetings to gather feedback from nearby residents, for being situated in critically underserved communities, and other detailed criteria [19]. There was also no requirement to match funds which might otherwise have put communities with less fiscal capacity at a disadvantage.

**Fig 1:**
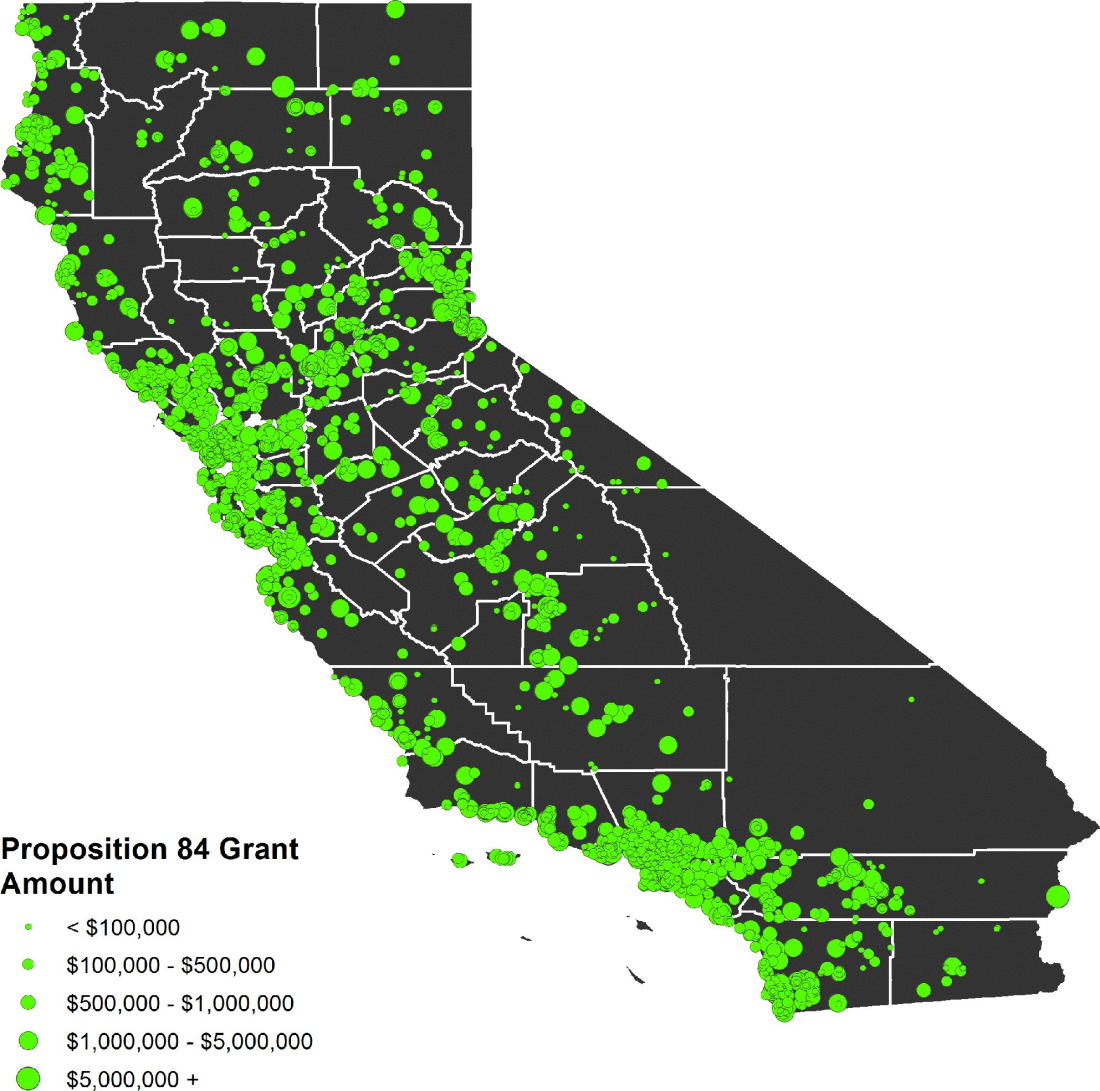
Location of all grants determined to have a local impact. Size corresponds to grant amount. Grants are heavily concentrated in population centers like Los Angeles and the San Francisco Bay Area, but were also disbursed throughout the Central Valley, Sierra Nevada, and along the coast. Geographic data from U.S. Census Bureau [20].

The location of all local grants for the three prioritized chapters are mapped in Fig 1A–1D.

## Methods

To assess how Proposition 84 funds were spent with regard to need and equity, we analyzed Proposition 84 spending at the level of census block groups (ranging in area from 0.015 to 16,000 km^2^). The underlying database was compiled by GreenInfo Network using grant information from the California Natural Resources Agency. This database included 2,152 projects for which a local footprint could be identified, thus enabling an analysis of the communities that benefited. Planning, technology, and large regional grants where a distinct local impact could not be identified were not included in this analysis, because we could not assign them to particular census blocks. Such projects include the construction of a website portal for the California Stormwater Quality Association, the creation of an urban greening plan for the entire city of San Diego, and a construction feasibility study for Madera County. Large regional water projects, and all projects funded under Chapter 4, “Statewide Water Planning and Design” were also considered non-local and thus excluded from our analysis. While some of these grants provide local benefits, it was impractical to verify where these benefits accrued from the information provided, in contrast to grants where a particular location for a project was identified. Grant funds were administered by 17 different agencies using different procedures and guidelines for soliciting and selecting projects for funding. While we were able to access a comprehensive database of projects that were funded, there is no comprehensive records of projects that were not funded. Therefore, we focused our analysis only on funded projects.

Altogether, the 2,152 projects with local impacts that we analyzed accounted for $2 billion of the total spending under Proposition 84. We added data concerning several pertinent environmental and socioeconomic attributes associated with each census block group (see Table 2). These quantitative attributes allow us to examine whether California block groups that benefited from Proposition 84 projects differed from those that did not. Altogether, there are 23,212 block groups in California, of which 1,242 received Proposition 84 project funding with an identifiable local impact.

**Table 2:** Variables and sources used in the analyses.

	Source
Median household income (MHI)	American Community Survey 2009–13, U.S. Census Bureau [21]
Population density	American Community Survey 2009–13, U.S. Census Bureau [22]
Urban coverage	TIGER 2013 Urban Areas, U.S. Census Bureau [23]. Coverage calculated as the percent of a block group that is covered by a Census urban area
Park space per capita	California Protected Areas Database 2015a, GreenInfo Network [24]
CES pollution exposure measures	CalEnviroScreen (CES) 2.0, California Office of Environmental Health Hazard Assessment [25]
Population change	2000 Census of Population and Housing, U.S. Census Bureau; standardized to 2010 Census Bureau geography by the National Historical Geographic Information System [26]

We used environmental data from CalEnviroScreen (CES), a California-wide tool to assess at-risk communities. However, we did not use the entire composite CES index score, because it contains a large array of sub-indicators not relevant for this study. Instead, we used only the pollution burden components, which include measures for drinking water and potential groundwater contamination, impaired water bodies, levels of ozone and particulate matter, pesticide use, hazardous waste and cleanup sites, toxic releases, and traffic density. The result is a ranking of block groups according to pollution burdens that could potentially harm the health of residents.

Using the data in Table 2, we asked whether funding favored areas based on socioeconomic characteristics, urban demographics, a shortage of park space per capita, or environmental pollution burdens. We did this analysis for each chapter separately since the chapters differed in their intent, language, and the specificity of guidance regarding priorities. The analysis itself was conducted in two ways. First, we used hurdle models to see if the variables in Table 2 could predict receipt of grants or amount of funding. The Proposition 84 spending data contain a large number of zeroes because the vast majority (94%) of block groups did not receive any grants. While this presents problems for conventional linear models, we can model this type of data with a hurdle or two-part model. The “hurdle” is represented by a probit model that predicts the receipt of a grant based on median household income [27]. We use median household income as the predictor for grant receipt because successfully writing a grant proposal is linked to the resources available to a community which is likely reflected in household incomes [3,4]. The second part is a truncated linear regression model that predicts amount of funding conditional on passing the initial hurdle of receiving a grant. We model grant funding using predictor variables that capture need and environmental inequity. In addition to green space deficits and pollution, we include measures on population density and urban coverage to understand how well funding was disbursed to urban population centers. Taken together, the hurdle model allows us to evaluate how funding was awarded along attributes of need in California communities.

The second part of the analysis was contrasting funded to unfunded block groups to evaluate how well all chapters funded low-income communities and how well the two parks chapters in particular (Chapters 8 and 9) funded park-poor urban communities. We used the same predictor variables for both Chapter 8 and Chapter 9, even though Chapter 8 did not specifically prioritize low-income areas. This allowed us to compare how these two chapters served need, recognizing that the chapters differed in the specificity and implications of their language. For continuous variables where the distribution of sample means met the normality assumptions, we conducted a two-sample t-test contrasting funded to unfunded block groups; if there is no difference between the means of these two groups, then there is no evidence of targeting. For park-space per capita, which did not meet the assumptions necessary for a t-test, we conducted a chi-squared goodness of fit test between funded and unfunded block groups using binned frequency data. For each test, unfunded block groups were comprised of all block groups that did not receive funding from a given chapter, but may or may not have received funding from other chapters.

## Results

**Fig 2:**
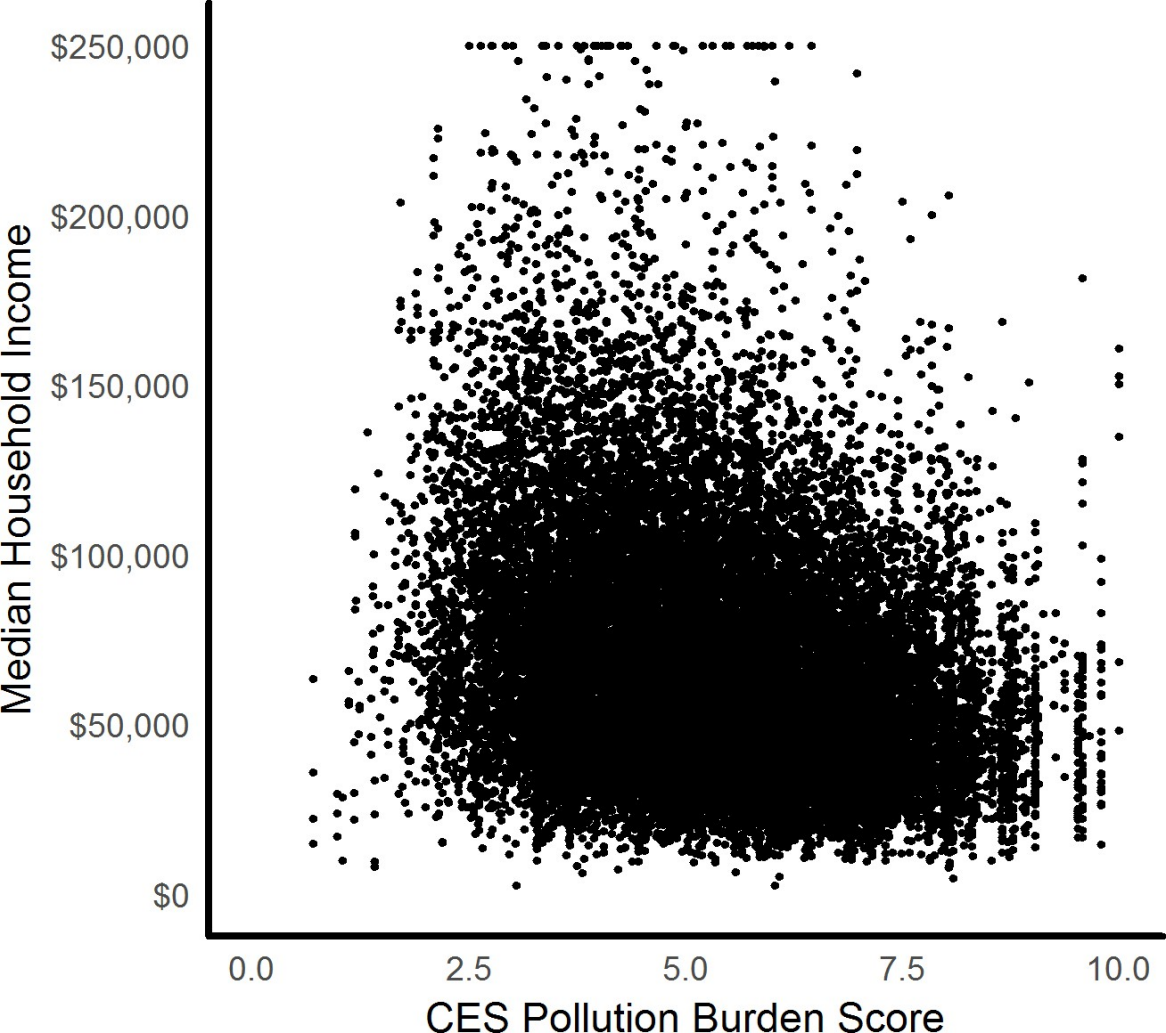
Median household income and CES pollution burden scores for all 23,312 California block groups (r = -0.29). Though low pollution burdens are present at all socioeconomic levels, greater pollution burdens are largely constrained to low-income block groups.

In California, pollution burdens are negatively correlated with median household income such that higher income communities are slightly less likely to suffer high pollution burdens (Fig 2).

Proposition 84 funding was distributed to block groups across all income levels. All chapters except Chapter 7 funded more grants in block groups at or below the average median household income than above, with Chapter 9 exhibiting the highest targeting of low-income block groups (Table 3).

**Table 3:** Funding and grants in block groups above and below average median household income. All chapters awarded more grants and funding to block groups below the average median household income, with the exception of Chapter 7.

Chapter	Spending and number of grants in block groups below average median household income	Spending and number of grants in block groups above average median household income
Chapter 2	$105,123,711 126	$37,577,886 47
Chapter 3	$154,049,689 25	$28,414,586 11
Chapter 5	$349,716,208 370	$174,393,869 224
Chapter 6	$224,204,698 139	$74,781,676 82
Chapter 7	$67,036,158 85	$93,920,423 164
Chapter 8	$127,090,114 352	$108,704,674 219
Chapter 9	$378,631,864 254	$40,046,527 54
Total	$1,405,852,442 1,351	$557,839,641 801

Across all block groups, those that received Proposition 84 funding had moderately lower incomes than unfunded block groups (Table 4). Chapters 2 and 9 stand out as being most effective at funding those communities on the lower scale of income, whereas Chapter 7 funded higher income block groups on average. Chapter 7 funded coastal protection projects, but within the subset of coastal block groups (n = 676), funded block groups had lower median household income on average ($71,856) compared to unfunded block groups ($84,748), though still higher than the average for all block groups within the state ($67,690). For Chapters 3, 4 and 8, there was no significant income difference between funded and unfunded block groups.

**Table 4:** Mean median household income for funded and unfunded block groups, by chapter. Differences between funded and unfunded income was tested for significance using two-tailed t-tests.

Chapters	Average median household income in funded block groups	Average median household income in unfunded block groups
All Chapters**	$62,346	$68,203
Chapter 2**	$59,811	$67,907
Chapter 3	*NS*	*NS*
Chapter 4	*NS*	*NS*
Chapter 5**	$62,249	$67,838
Chapter 6*	$62,772	$67,724
Chapter 7**	$80,915	$67,529
Chapter 8	*NS*	*NS*
Chapter 9**	$47,919	$68,080

* p < 0.05

** p < 0.01

NS identifies chapters for which the mean value for funded block groups did not differ significantly from the mean value of unfunded block groups

With clear differences in the pollution burdens of high and low-income block groups, and in the incomes of funded and unfunded block groups, we used a hurdle model to see if income could predict receipt of grants, and if population density, urban coverage, park space per capita, and pollution burdens could predict funding amount conditional on receipt. The hurdle model revealed biases that were significant but had very small effect sizes. (Table 5). The probability of a block group receiving a grant from any chapter decreased slightly with median household income, with block groups of $50,000 median income having a 1.5% greater probability of receiving a grant than those of $150,000. The linear models yielded some small effects of the covariates on grant size. In particular, greater population density predicted slightly more funding from all grants, and Chapter 2 grants in particular, and higher pollution scores were associated with more funding from Chapter 9 grants. For all other chapters, the hurdle model had poor predictive power with wide margins of error, and did not reveal significant effects on funding from the covariates (S1 Fig). Surprisingly, even park space per capita was not a strong predictor of funding amount for Chapter 9 for which park-poor communities were a priority.

**Table 5:** Hurdle model results. Coefficient are estimated for grant selection and for magnitude of funding. The top values are coefficient estimates, the bottom values are standard errors.

Probability of receiving a grant (logit)	All Chapters	Chapter 2	Chapter 3	Chapter 5	Chapter 6	Chapter 7	Chapter 8	Chapter 9
MHHI ($10,000)	-0.03 0.00	0.00 1695.20	0.00 1255.46	0.00 1337.71	0.00 1,912.71	0.00 1,046.21	0.00 1616.42	0.00 1972.73
**Magnitude of Funding (Gaussian)**								
Population Density	***63.30 57.3	219.42 57.31	2,793.32 2,074.70	69.60 306.51	-564.64 2,229.26	54.66 97.67	199.08 140.86	30.78 17.12
Percent Urban	-7,506.37* 3,125.85	2,151.03 3,125.85	46,998.48 71,003.42	-14,878.96 11,268.48	-16,184.73 18,901.60	-3,941.43 3,159.94	3,166.20 3,709.65	478.63 4,147.30
Park Acres per 1000 People	-0.39 1.40	- 0.83 1.40	-255.48 512.85	- 2.18 2.07	**9.07 2.93	- 1.24 3.18	-0.01 0.25	-2.34 4.35
Pollution Burden Score	*18,3151.40 74,678.52	127,843.70 74,678.52	-165,852.10 1,940,278.00	299,866.70 213,025.20	-11,030.34 179,894.30	18,740.86 62,791.01	618.60 67,511.45	**175,753.20 65,904.17

*** p > 0.001

** p > 0.01

* p > 0.05

Finally, we examined the two park chapters of Proposition 84, Chapters 8 and 9, and compared how well they funded park-poor urban communities. Our analysis revealed several strong biases (Table 6). Grants for Chapter 8, “Parks and Nature Education Facilities,” were preferentially awarded to sparsely populated rural areas with dramatically more park space per capita than unfunded block groups. In contrast, grants for Chapter 9, “Sustainable Communities and Climate Change Reduction” favored areas with very little park space per capita. Block groups that received these grants did not differ significantly from unfunded block groups in terms of urban coverage and population density, but because most block groups in California are urban and densely populated, this does not necessarily signify a departure from the chapter’s stated intent. Neither Chapter 8 nor Chapter 9 favored areas with greater population growth.

**Table 6:** Evaluating funding from Chapters 8 and 9 to urban communities. Values are expressed as the ratio between the average value of funded and unfunded block groups for a given variable. Chapter 9.

	Ratio of funded to unfunded block groups			
Variables	Population density*	Urban coverage*	Park space per capita†	Population growth 2000–2010*
Chapter 8: “Parks and Nature Education Facilities”	0.15	0.35	25.19	*NS*
Chapter 9: “Sustainable Communities and Climate Change Reduction”	*NS*	*NS*	0.55	*NS*

* Significance tested with two-sample, two-tailed t-test

† Significance tested with chi-squared test using binned frequency data

*NS* identifies a variable where the mean value for block groups funded by a given chapter did not differ significantly from the mean value of unfunded block groups

## Discussion

This paper examined the disbursement of funds from a major California environmental ballot measure with respect to household income, pollution, and other variables related to urban environmental justice. We found that while a majority of the proposition’s chapters did on average fund projects in lower income areas, some did not. In particular, funding for coastal protection and state parks under Chapters 7 and 8 went to wealthier areas. Within the subset of only coastal block groups, Chapter 7 grants went to those with lower median household income on average, though still higher than the average across for block groups across the state. This is a function of real estate values–coastal block groups are generally wealthier and have higher property costs compared to inland block groups. On the other hand, Chapters 2 and 9, which had language clearly prioritizing low-income communities, did very well at targeting funding to low-income block groups.

The hurdle models revealed that the probability of a block group receiving a grant decreased slightly with median household income. However, income could not predict receipt of grants from individual chapters, possibly because there were so few block groups funded by any given chapter compared to the number of unfunded block groups in the state. Given that a block group received a grant, very few of the covariates of interest could predict the size of that grant. In other words, larger grants did not seem to necessarily go to the areas that were the most urban, the most park-poor, or the most polluted. There were two exceptions to this–greater population density was associated with larger Chapter 2 grants and worse pollution was associated with larger Chapter 9 grants. Surprisingly, park space and income were not good predictors of funding for Chapter 9 grants which specifically targeted disadvantaged urban communities. Overall, the receipt and size of grants could not be predicted even with a two-part model, possibly because of the sheer number of unfunded block groups and other factors that may be important to the grant award process.

When we examined the two park chapters, Chapters 8 and 9, we found that Chapter 9 was exceptionally successful at meeting its stated priorities of funding park projects in lower-income urban communities that lack park space. Chapter 8, though not intended to fulfill the same role as Chapter 9 projects, nevertheless had a stated priority to reflect growing population centers. This goal of targeting areas of population growth was not met, and grants were awarded overwhelmingly to sparsely populated rural areas. It should be noted that acres per 1,000 residents and population density are blunt measures that may not tell the whole story. More detailed analyses that distinguished multi-family from single family housing, or that used actual satellite images, or that reflected the extent to which neighborhoods even wanted more parks would be the next steps in this research.

A review of grant objectives reveals that nearly 77% of funds from Chapter 9 went towards construction of new parks, compared to less than 5% of Chapter 8 funds. Thus, the two parks chapters served different needs in different populations, despite both intending to expand parks in the state. While our hurdle model revealed that lower income block groups were more likely to be awarded grants overall, the mechanisms behind submitting and being approved for a grant were not investigated in this study. As previous studies have noted, competitive grants tend to favor communities with the resources to submit strong proposals, or any proposals at all [8,9]. These factors, in addition to the social and economic capital required to obtain space and approval for parks, are an area of exploration for future research.

One question that we tried to answer in this paper was if the funding disbursement matched the intention of the ballot measure in the sections of the ballot that did state a preference for disadvantaged communities. There is also the broader question of how well the funds remedied inequities in environmental amenities and environmental quality overall even if the language did not specify such a goal. An argument can and has been made that public investments in the environment, particularly urban sustainability or park projects, should prioritize low-income communities because those communities often bear greater pollution burdens and green space deficits and have less local fiscal capacity to fund projects [10,28,29].

Evaluating how well public environmental investments serve low-income communities is important not only for gauging the commitment to environmental justice, but also for informing how future legislation and ballot measures might be written. In the case of Proposition 84, language might have been an important factor. Chapter 8 was written with the somewhat vague goal of expanding parks to reflect growing and shifting population centers, yet funding tended to go to rural areas with an abundance of park space, no significant population growth, and no significant difference in income from unfunded areas. In contrast, Chapter 2 prioritized low-income communities amongst other criteria and awarded nearly 3 times as much grant money to block groups below the average median household income. And finally, Chapter 9, with the strictest language explicitly constraining funding to underserved park-poor or low-income) communities, awarded over 9 times as much funding to low-income block groups.

In addition to language, there are likely systemic reasons that disadvantaged communities were not funded more in some parts of Proposition 84. A critical caveat for any analysis attempting to evaluate awards from a competitive grant process is that there is rarely, if ever, a database of grant applications that were rejected. This leads one to ask if poorer communities received fewer grants because they simply did not submit grants, or perhaps their proposals were not as professionally prepared. It is essential that a data base of successful and unsuccessful proposals is maintained, because only with those data could one suggest more precise remedies to the problem of underserving communities with public funds. If communities are not submitting grants, efforts at greater education and outreach from public agencies about funding opportunities or grant-writing workshops might be needed. For instance, the Department of Parks and Recreation held a number of technical workshops for writing AB 31 grants [19]. But if disbursement agencies are not awarding grants to these communities even if they apply, then a more transparent award process is needed with greater efforts made towards serving these areas.

Proposition 84 attempted to serve many different needs when it came to environmental infrastructure, with mixed results when it came to serving the urban communities most in need. This is perhaps not surprising, since the individuals and organizations that wrote the proposition and funded the campaign for this ballot measure tended to represent traditional conservation organizations and did not include environmental justice groups, aside from AB 31. As previous research has shown, nonprofits often act as powerful political actors in determining, through contributions, what benefits are received from ballot measures [30]. In particular, the two top funders of Proposition 84 were The Nature Conservancy $3,549,920) and California Conservation Action Fund $1,574,074) [31]. The lesson for public ballot measures is clear: the “devil is in the details” of the text. Since equity historically has not been a focus of these conservation groups, it may come as little surprise that most chapters of Proposition 84 did not contain stronger and more precise language directing money toward lower income or communities deprived of nature opportunities. However, there is some indication that environmental initiative sponsors and legislators in California are increasingly following the model established by Chapter 9 and AB 31 in Proposition 84, and are explicitly directing funds to communities in most need [32,33]. Ultimately, we should expect money to flow to these areas of greatest need only if they are prioritized, given access to grant-writing opportunities, and including in the writing of the measure.

## Supporting information

S1 File Data used for the analysis. (CSV)

S1 Fig Predicted probabilities of receiving a grant. (PDF)

S2 Fig Predicted magnitude of grant funding (All Chapters and Chapters 2, 3, 5). Predicted grant funding for selected variables holding all others constant.
(PDF)

S3 Fig Predicted magnitude of grant funding (Chapters 6, 7, 8, 9). Predicted grant funding for selected variables holding all others constant.
(PDF)

## References

[1] Trust for Public Land. TPL LandVote Database [Internet]. 2015 [cited 4 May 2016]. Available: TPL LandVote Database

[2] Scarlett L. Citizens Vote Green: Approve $29 Billion in Land and Water Funding at the Ballot Box | Conservancy Talk. In: The Nature Conservancy [Internet]. 2014 [cited 7 Aug 2017]. Available: https://blog.nature.org/conservancy/2014/11/05/citizens-vote-green-approve-29-billion-in-land-and-water-funding-at-the-ballot-box/

[3] Wolch J, Wilson JP, Fehrenbach J. Parks and Park Funding in Los Angeles: An Equity-Mapping Analysis. Urban Geogr. Taylor & Francis Group; 2005;26: 4–35. 10.2747/0272-3638.26.1.4

[4] Walls M. Parks and Recreation in the United States: Local Park Systems [Internet]. Washington, DC; 2009. Available: http://www.mparks.org/Portals/0/Resource-Center/Justifying Parks and Recreation/Economic Impact/ResourcesfortheFuture-PandRintheUS-LocalParks.pdf

[5] Marvier M, Wong H. Resurrecting the conservation movement. J Environ Stud Sci. Springer-Verlag; 2012;2: 291–295. 10.1007/s13412-012-0096-6

[6] Sze J, Gambirazzio G, Karner A, Rowan D, London J, Niemeier D. Best in Show? Climate and Environmental Justice Policy in California. Environ Justice. Mary Ann Liebert, Inc. 140 Huguenot Street, 3rd Floor New Rochelle, NY 10801 USA; 2009;2: 179–184. 10.1089/env.2009.0028

[7] Villamagna AM, Mogollón B, Angermeier PL. Inequity in ecosystem service delivery: socioeconomic gaps in the public-private conservation network. Ecol Soc. The Resilience Alliance; 2017;22: art36. 10.5751/ES-09021-220136

[8] Sister C, Wolch J, Wilson J. Got green? addressing environmental justice in park provision. GeoJournal. Springer Netherlands; 2010;75: 229–248. 10.1007/s10708-009-9303-8

[9] Loukaitou-Sideris A. Urban Form and Social Context: Cultural Differentiation in the Uses of Urban Parks. J Plan Educ Res. Sage PublicationsSage CA: Thousand Oaks, CA; 1995;14: 89–102. 10.1177/0739456X9501400202

[10] Joassart-Marcelli P. Leveling the Playing Field? Urban Disparities in Funding for Local Parks and Recreation in the Los Angeles Region. Environ Plan A. SAGE PublicationsSage UK: London, England; 2010;42: 1174–1192. 10.1068/a42198

[11] Pincetl S. Nonprofits and Park Provision in Los Angeles: An Exploration of the Rise of Governance Approaches to the Provision of Local Services*. Soc Sci Q. Wiley/Blackwell (10.1111); 2003;84: 979–1001. 10.1046/j.0038-4941.2003.08404019.x

[12] García R, Rawson Z, Yellott M, Zaldaña C. Healthy Parks, Schools and Communities for All: Park Development and Community Revitalization [Internet]. Los Angeles; 2009. Available: www.cityprojectca.org

[13] Institute of Governmental Studies. Proposition 84 [Internet]. Berkeley, CA: University of California, Berkeley; 2006. Available: https://igs.berkeley.edu/library/elections/proposition-84

[14] Smith-Heisters S, Summers AB. Analysis of California’s Propositions IE and 84: Funding the State’s Water and Flood Control Infrastructure [Internet]. Los Angeles; 2006. Available: http://reason.org/files/9c69395abc3615f6a55ef12355dcbd18.pdf

[15] Lewis WS. Ballot-Box Environmentalism across the Golden State: How Geography Influences California Voters’ Demand for Environmental Public Goods. Pomona Sr Theses. 2016; Available: https://scholarship.claremont.edu/pomona_theses/149

[16] Safe Drinking Water, Water Quality and Supply, Flood Control, River and Coastal Protection Bond Act of 2006 [Internet]. 84 2006 p. 14. Available: https://www.parks.ca.gov/pages/1008/files/prop_84_text.pdf

[17] de León K. Statewide Park Development and Community Revitalization Act of 2008 [Internet]. 31 Sacramento, CA: California Assembly; 2008 p. 8. Available: https://www.parks.ca.gov/pages/1008/files/ab_31_bill_9-2008_chaptered.pdf

[18] Park funds for park poor and income poor communities -Prop 84 and AB 31 standards are working! In: The City Project [Internet]. 2014 [cited 29 Oct 2018]. Available: https://www.cityprojectca.org/blog/archives/32075

[19] California Department of Parks and Recreation. Application Guide for the Statewide Park Development and Community Revitalization Program of 2008 [Internet]. Sacramento; 2009. Available: www.parks.ca.gov/grants.

[20] United States Census Bureau. 2013 TIGER/Line Shapefiles [Internet]. 2013. Available: https://www.census.gov/cgi-bin/geo/shapefiles/index.php

[21] United States Census Bureau. S1903 Median Income in the Past 12 Months (In 2013 Inflation-Adjusted Dollars) [Internet]. American FactFinder: 2009–2013 American Community Survey 5-Year Estimates: Demographic and Housing Estimates. 2013. Available: http://factfinder.census.gov

[22] United States Census Bureau. B00001 Unweighted Sample Count of Total Population. In: American FactFinder: 2009–2013 American Community Survey 5-Year Estimates: Demographic and Housing Estimates [Internet]. 2013. Available: http://factfinder.census.gov

[23] United States Census Bureau. 2013 Census Urban Areas. In: 2013 TIGER/Line Shapefile [Internet]. 2013. Available: https://www.census.gov/geo/maps-data/data/tiger.html

[24] GreenInfo Network. California Protected Areas Data Portal 2015a [Internet]. 2015. Available: http://www.calands.org/

[25] Office of Environmental Health Hazard Assessment. CalEnviroScreen Version 2.0 [Internet]. 2015 [cited 7 Aug 2017]. Available: https://oehha.ca.gov/calenviroscreen/report/calenviroscreen-version-20

[26] Minnesota Population Center. National Historical Geographic Information System: Version 11.0 [Internet]. Minneapolis, MN: University of Minnesota; 2016. 10.18128/D050.V11.0

[27] Henningsen A, Toomet O. maxLik: A package for maximum likelihood estimation in R. Comput Stat. Springer-Verlag; 2011;26: 443–458. 10.1007/s00180-010-0217-1

[28] Bullard RD. Environmental Justice in the 21st Century: Race Still Matters. Phylon (1960-). Clark Atlanta University; 2001;49: 151 10.2307/3132626

[29] Heynen N, Perkins HA, Roy P. The Political Ecology of Uneven Urban Green Space The Impact of Political Economy on Race and Ethnicity in Producing Environmental Inequality in Milwaukee. Urban Aff Rev. 2006;42: 3–25. 10.1177/1078087406290729

[30] Pincetl S. Nonprofits and Park Provision in Los Angeles: An Exploration of the Rise of Governance Approaches to the Provision of Local Services*. Soc Sci Q. Blackwell Publishing; 2003;84: 979–1001. 10.1046/j.0038-4941.2003.08404019.x

[31] Cal-Access. Campaign finance—Water quality, safety and supply. Flood control. Natural resource protection. Park improvements. Bonds. Initiative statute. In: California Secretary of State [Internet]. 2006 [cited 24 Aug 2017]. Available: http://cal-access.sos.ca.gov/Campaign/Measures/Detail.aspx?id=1283864&session=2005

[32] Rosenhall L. California Capitol focuses on environmental injustice—but will it lead to real results? In: CALmatters [Internet]. 2017 [cited 30 Aug 2017]. Available: https://calmatters.org/articles/california-capitol-hones-environmental-injustice-will-focus-lead-real-results/

[33] Megerian C. In the battle over California climate policies, green projects are now in the hot seat. Los Angeles Times. Los Angeles; 13 Mar 2017. Available: http://www.latimes.com/politics/la-pol-ca-offsets-environmental-justice-20170313-story.html

