# Supplementary figures and images for: Assessing the flow to low-income urban areas of conservation and environmental funds approved by California’s Proposition 84
Source: PLoS One. 2019 Feb 7;14(2):e0211925. doi: 10.1371/journal.pone.0211925 (PMC6366786; doi:10.1371/journal.pone.0211925)

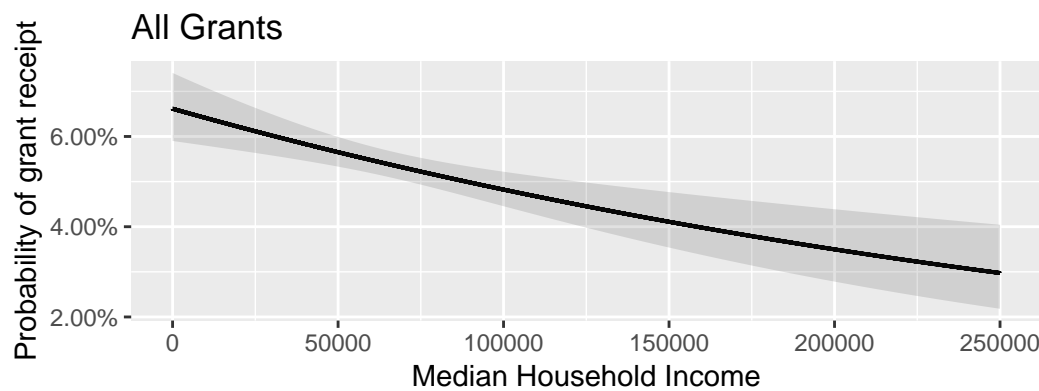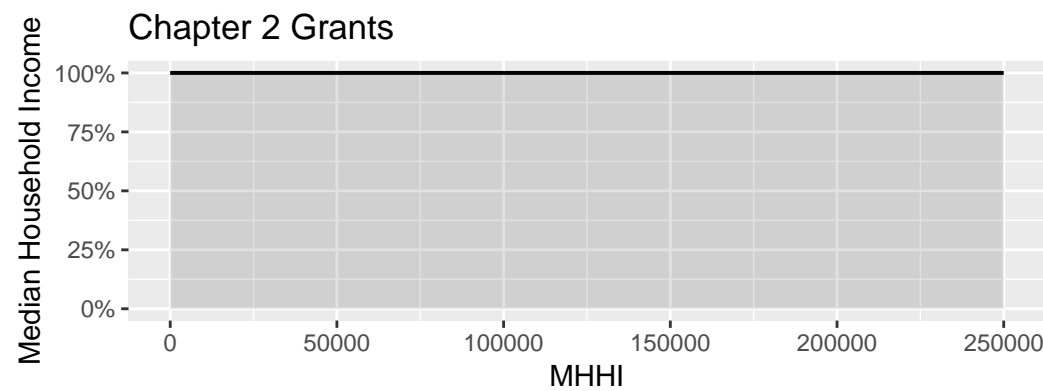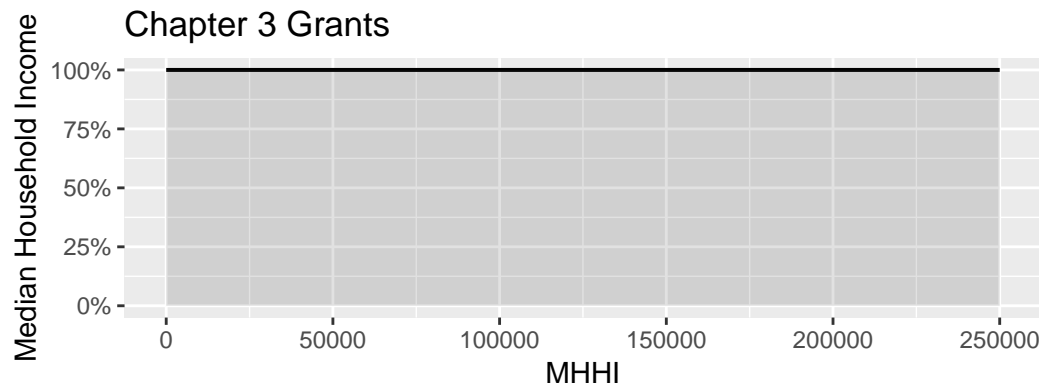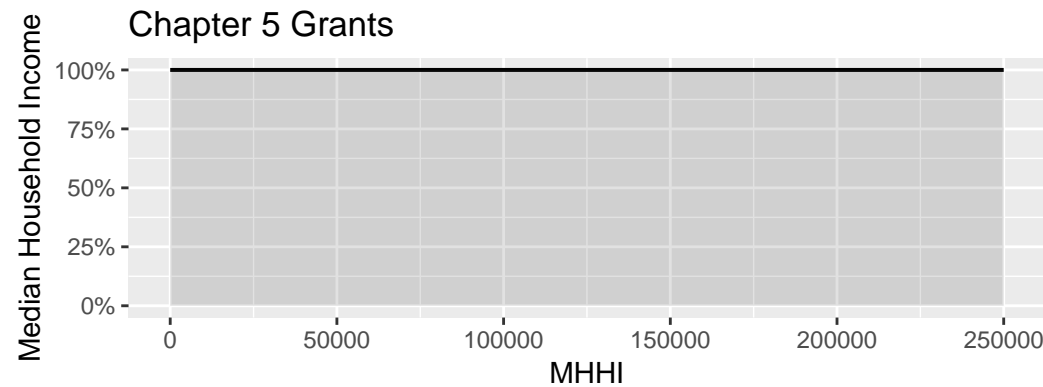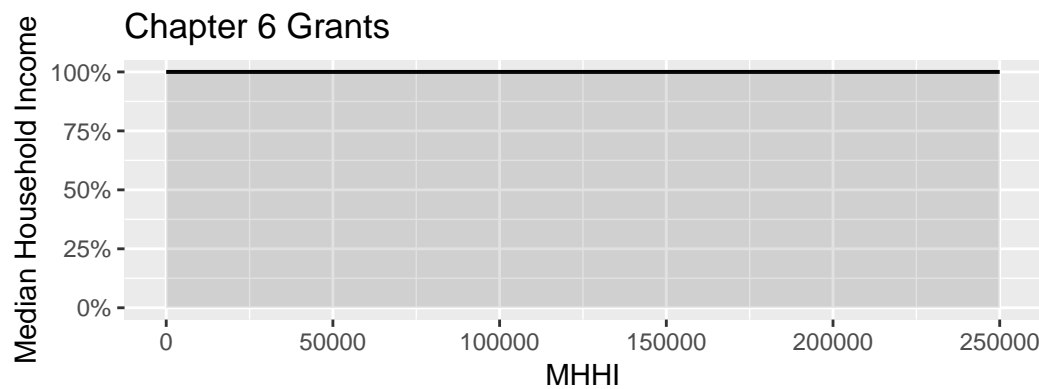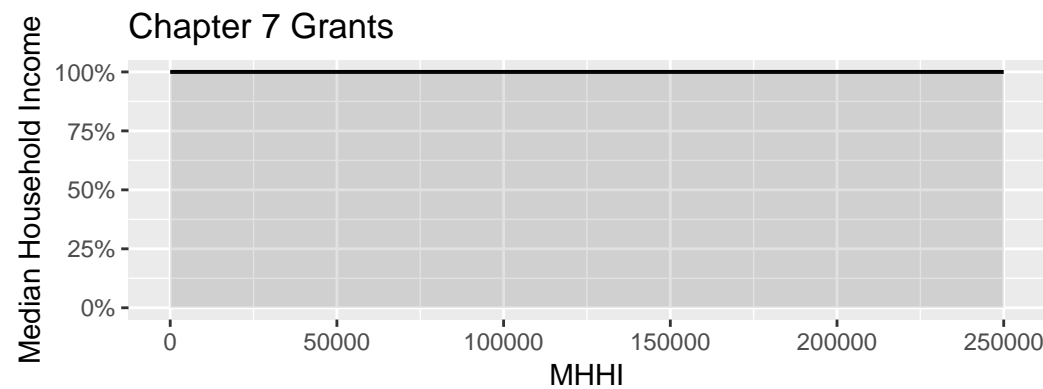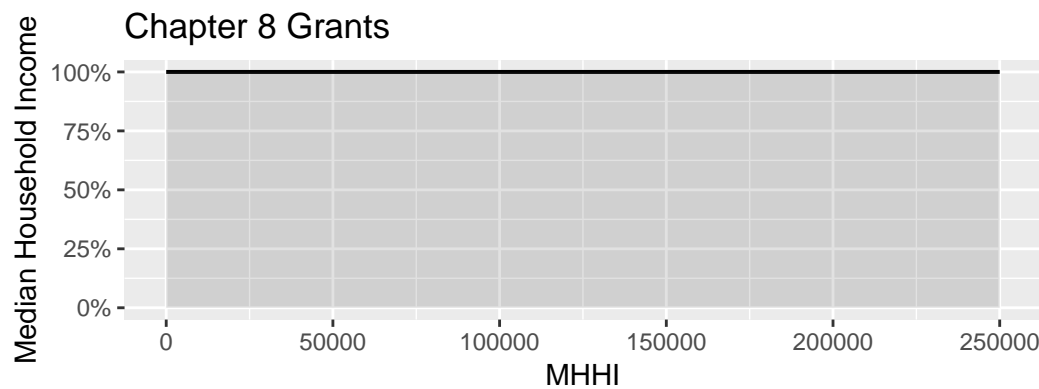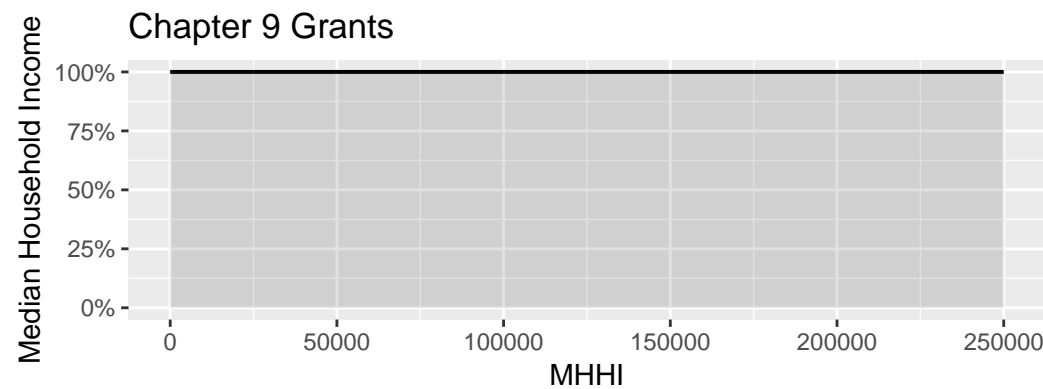

Supplement: S1 Fig — (PDF) [file pone.0211925.s002.pdf]

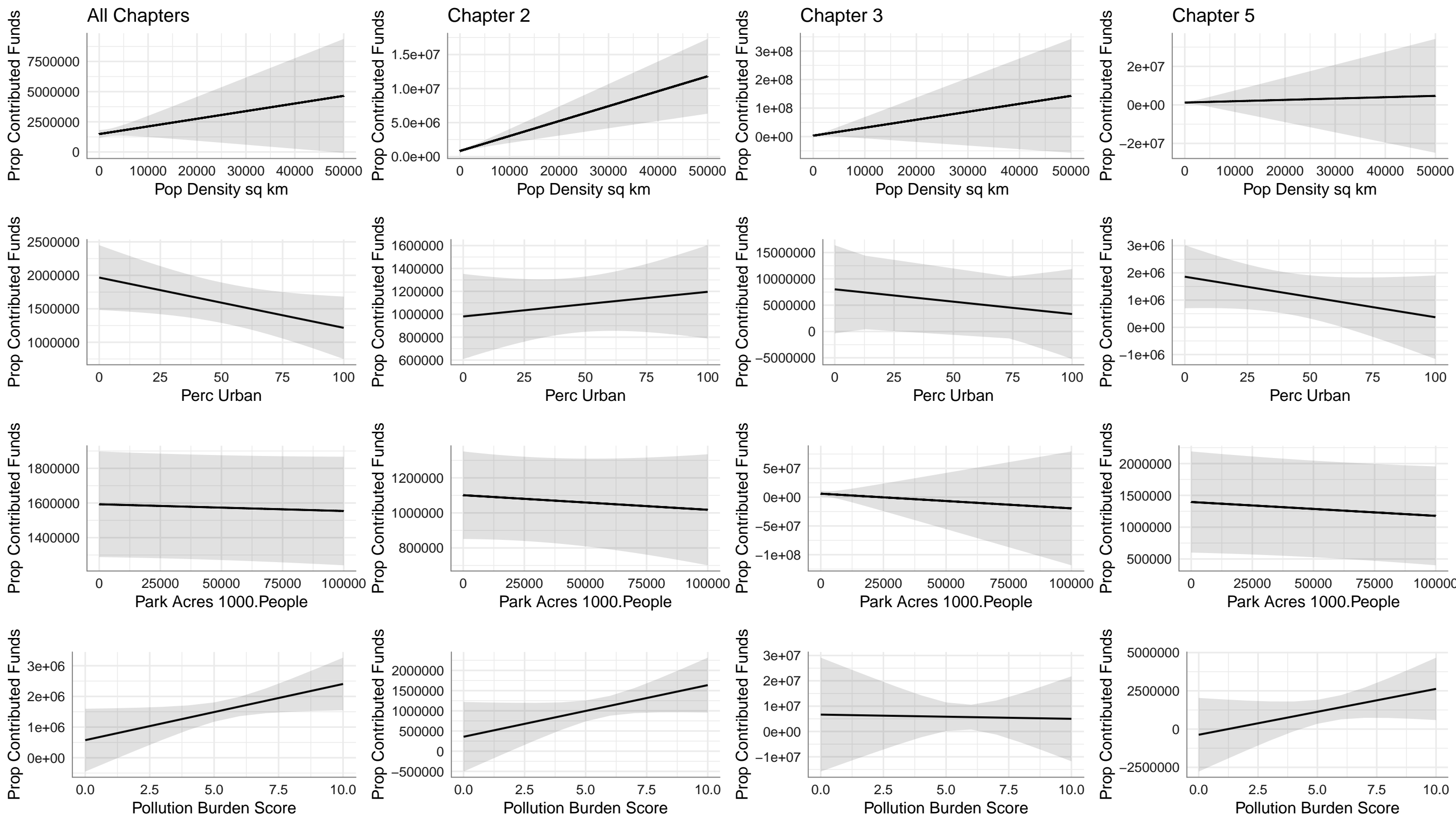

Supplement: S2 Fig — Predicted grant funding for selected variables holding all others constant. (PDF) [file pone.0211925.s003.pdf]

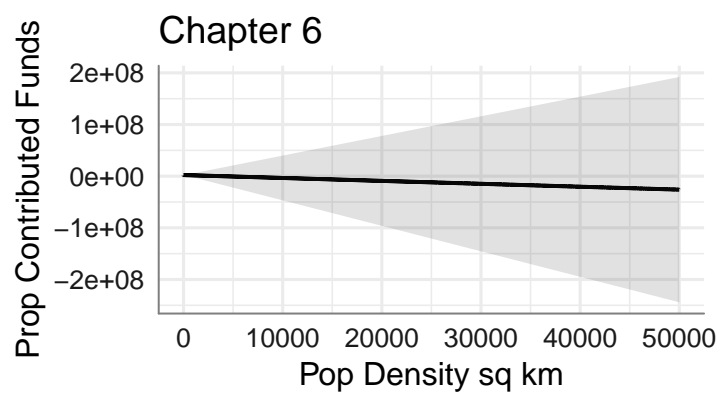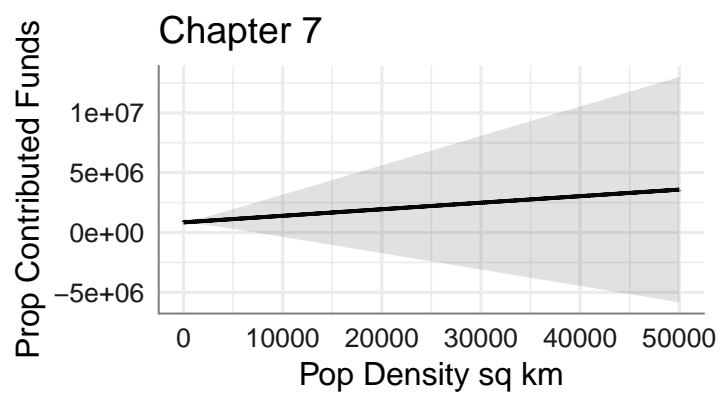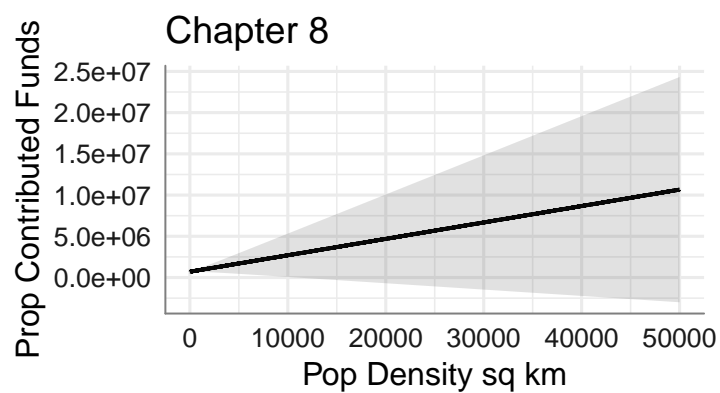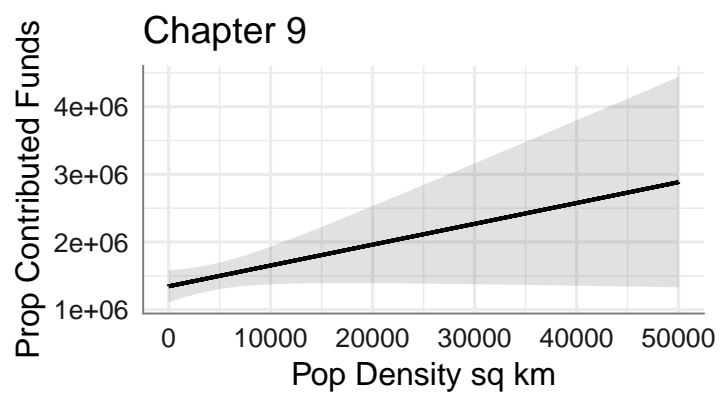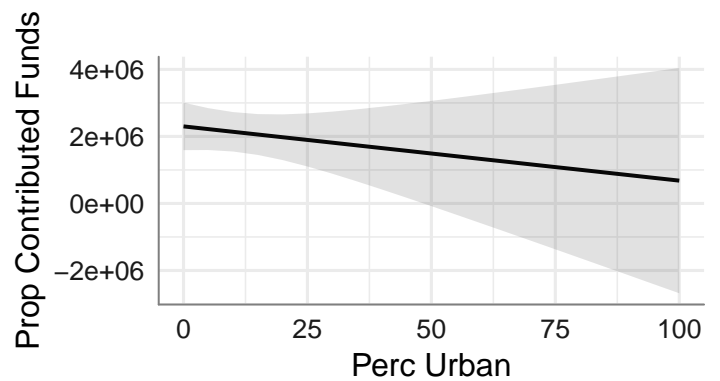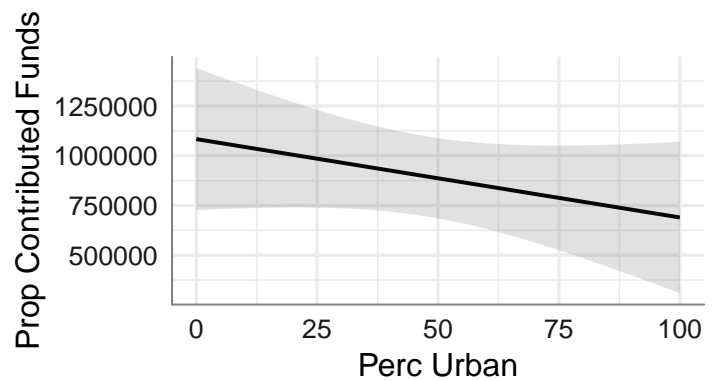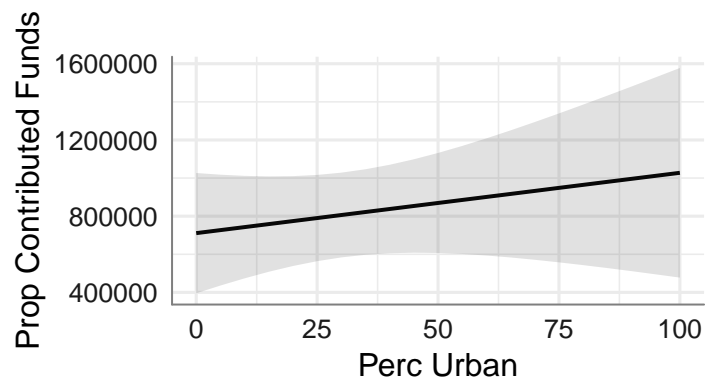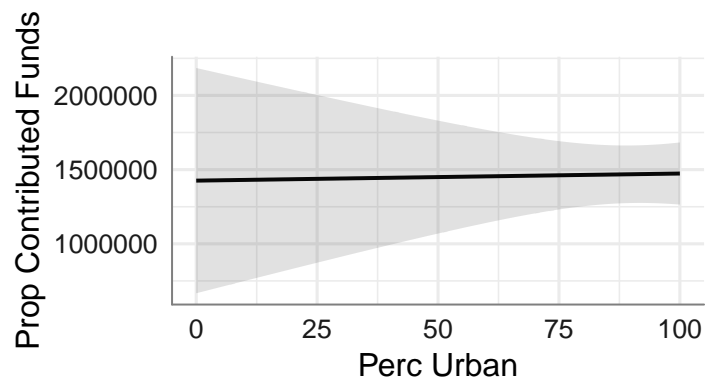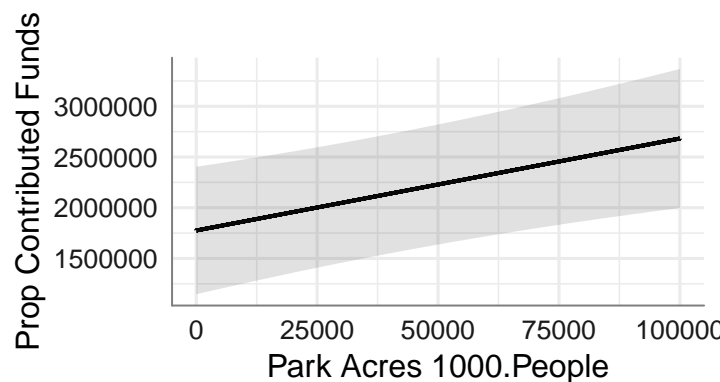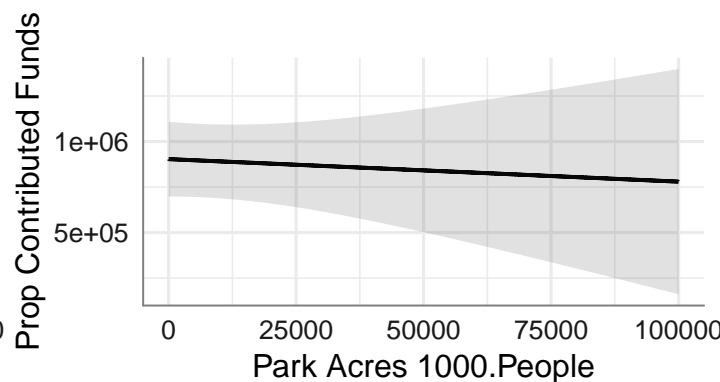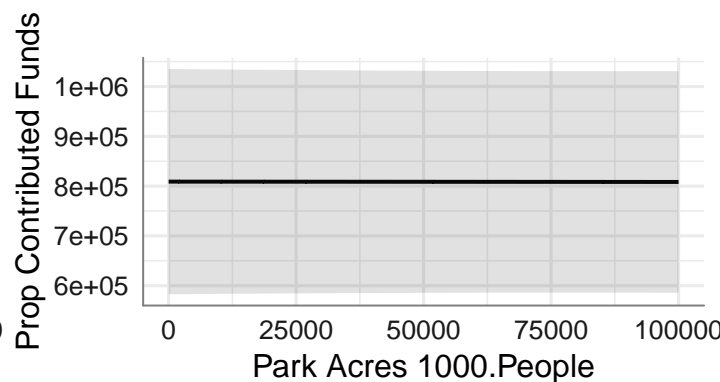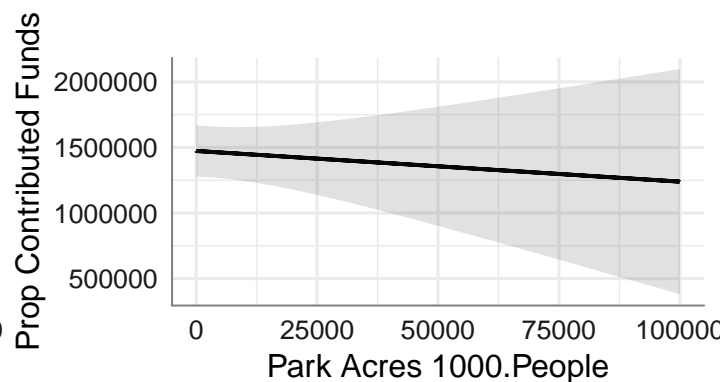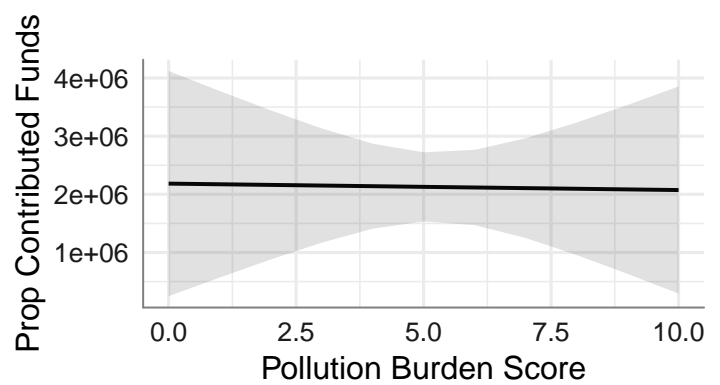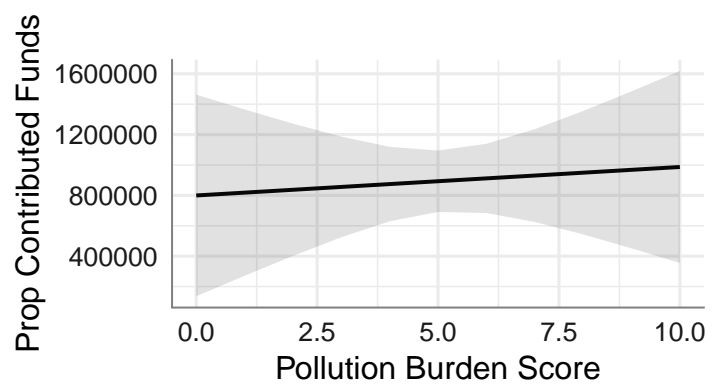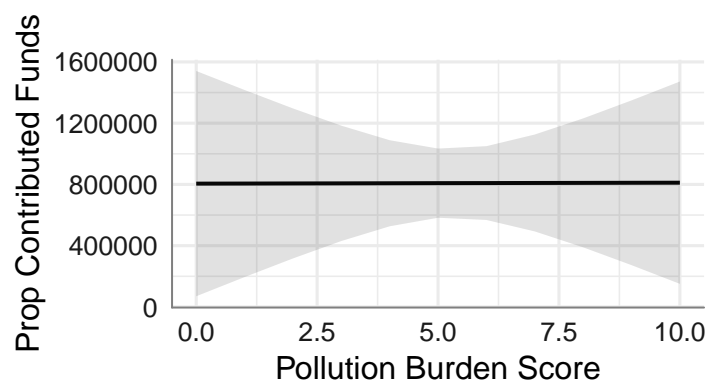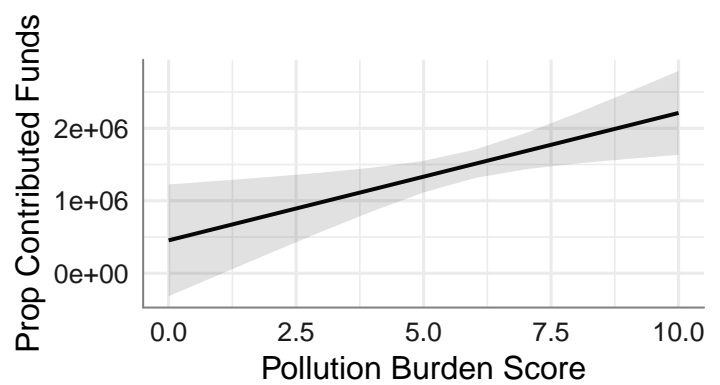

Supplement: S3 Fig — Predicted grant funding for selected variables holding all others constant. (PDF) [file pone.0211925.s004.pdf]
